# A novel method for screening the glutathione transferase inhibitors

**DOI:** 10.1186/1471-2091-10-6

**Published:** 2009-03-16

**Authors:** Zhijun Wang, Li Jin, Grzegorz Węgrzyn, Alicja Węgrzyn

**Affiliations:** 1 CAS-MPG Partner Institute for Computational Biology, Shanghai Institutes for Biological Sciences, Chinese Academy of Sciences, 200031, Shanghai, PR China; 2 MOE Key Laboratory of Contemporary Anthropology and Center for Evolutionary Biology, School of Life Sciences and Institutes of Biomedical Sciences, Fudan University, 200433, Shanghai, PR China; 3 Department of Molecular Biology, University of Gdańsk, 80-822 Gdańsk, Poland; 4 Laboratory of Molecular Biology (affiliated with the University of Gdańsk), Institute of Biochemistry and Biophysics, Polish Academy of Sciences, 80-822 Gdańsk, Poland

## Abstract

**Background:**

Glutathione transferases (GSTs) belong to the family of Phase II detoxification enzymes. GSTs catalyze the conjugation of glutathione to different endogenous and exogenous electrophilic compounds. Over-expression of GSTs was demonstrated in a number of different human cancer cells. It has been found that the resistance to many anticancer chemotherapeutics is directly correlated with the over-expression of GSTs. Therefore, it appears to be important to find new GST inhibitors to prevent the resistance of cells to anticancer drugs. In order to search for glutathione transferase (GST) inhibitors, a novel method was designed.

**Results:**

Our results showed that two fragments of GST, named F1 peptide (G**YW**KIKG**L**V) and F2 peptide (KW**R**NK**K**FELGLEFP**N**L), can significantly inhibit the GST activity. When these two fragments were compared with several known potent GST inhibitors, the order of inhibition efficiency (measured in reactions with 2,4-dinitrochlorobenzene (CDNB) and glutathione as substrates) was determined as follows: tannic acid > cibacron blue > F2 peptide > hematin > F1 peptide > ethacrynic acid. Moreover, the F1 peptide appeared to be a noncompetitive inhibitor of the GST-catalyzed reaction, while the F2 peptide was determined as a competitive inhibitor of this reaction.

**Conclusion:**

It appears that the F2 peptide can be used as a new potent specific GST inhibitor. It is proposed that the novel method, described in this report, might be useful for screening the inhibitors of not only GST but also other enzymes.

## Background

Glutathione transferase (GST) (EC 2.5.1.18) is a multifunctional enzyme, which protects cells against cytotoxic and genotoxic stresses. GST catalyzes the conjugation of cytotoxic agents to glutathione (γ-glutamyl-cysteinyl-glycine), producing less reactive chemical species. Changes in GST levels have been found to correlate with resistance to anticancer drugs through accelerated detoxification of these drugs' substrates [1-4].

Members of the GST family are present at relatively high concentrations in the cytosol of various mammalian tissues. Over-expression of GST isozymes has been reported in a number of different human cancers, when compared to the corresponding normal tissues [5,6]. A 2-fold increase in GST activity was found in lymphocytes from chronic lymphocytic leukemia (CLL) patients, who were resistant to chlorambucil, relative to lymphocytes from untreated CLL patients [7]. As GST isozymes are frequently up-regulated in many solid tumors and lymphomas, inhibition GST activity has become a new drug design concept [8-13]. These facts led to the search for and design of GST inhibitors, including their synthetic analogues and glutathione conjugates, however, most of the existing inhibitors are either too toxic to be used *in vivo *or are effective only *in vitro *[14,15].

Although several different GST inhibitors have been reported, to our knowledge, there are no reports on design of the GST inhibitors according to GST sequence. In this report, a novel, covering all gene fragments (CAGF), cloning method was used to screen the GST fragments which can bind to glutathione and form the inhibitory complexes. These inhibitory complexes act as modified substrate inhibitors or substrate homologues to inhibit the GST activity. The method described in this report should be suitable not only for development of novel drugs inhibiting the GST activity, but also for finding effective inhibitors in other enzyme-catalyzed reaction systems.

## Results

### Screening the GST inhibitors using the fragments of GST

**Figure 1:**
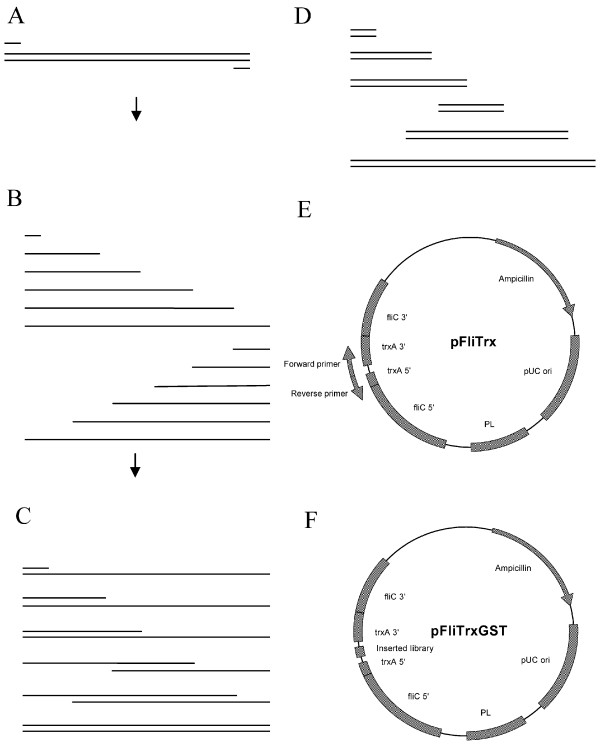
**Cloning all GST gene fragments into the plasmid DNA vector with the covering all gene fragments (CAGF) cloning method**. A): The gene fragments of GST, B): The amplification of GST fragments using the system containing ddNTP, which can terminate the amplification reaction, and produce the DNA sequences with the single base differences, thus, the reaction system can produce a large library of fragments with single base differences. C): The binding of amplified products, D): Digestion of the CAGF cloning products with Exonuclease VII to form the blunt-ended DNA fragments. E): Amplification of the whole pFliTrx plasmid with the primers FP2 and RP2, F): The linearized pFilTrx plasmid was linked with the DNA library of the gene encoding GST.

**Figure 2:**
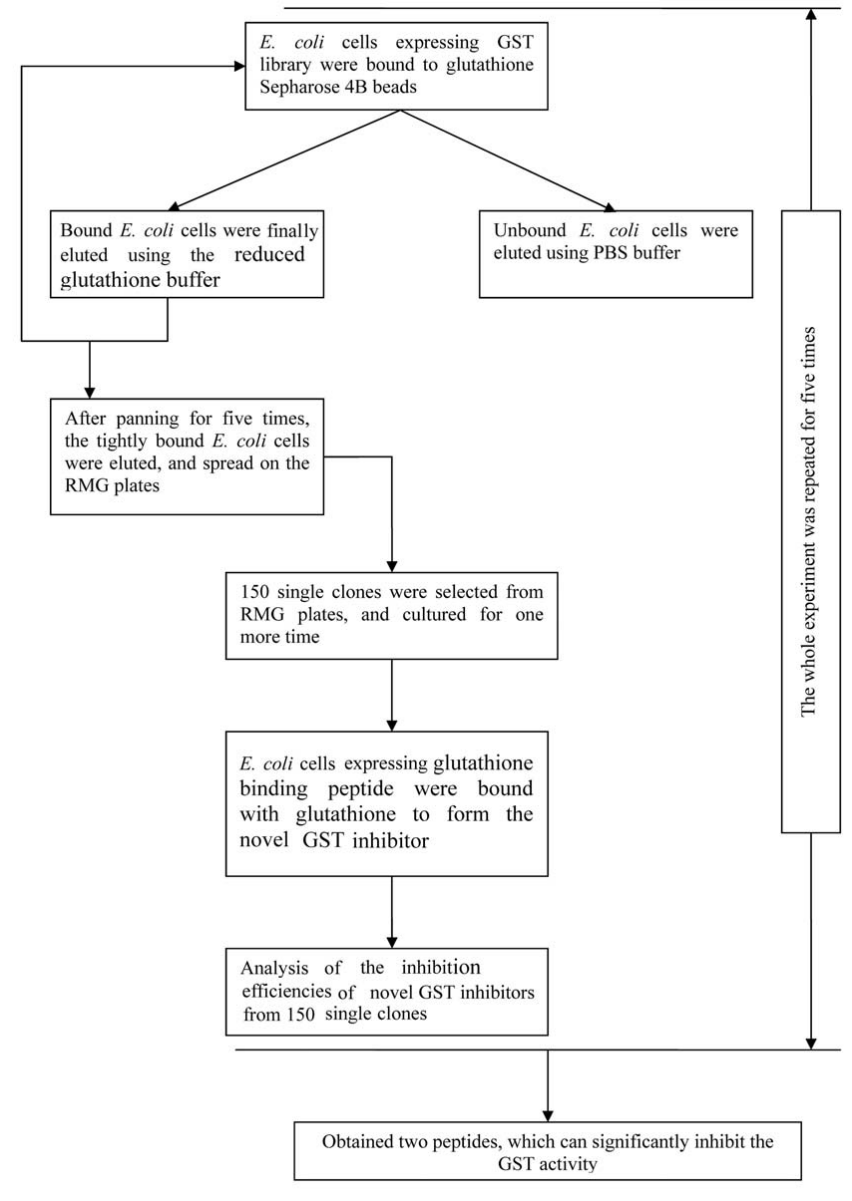
**The experimental procedure for screening the fragments of GST which can significantly inhibit GST activity**.

The scheme of the 'covering all gene fragments' (CAGF) cloning method is shown in Fig. 1, and the whole screening procedure is shown in Fig. 2. Following five-time panning procedure, as described in the Methods section, 150 positive clones, which can tightly bind to the glutathione Sepharose 4B beads, were picked up from the plates. The typical panning efficiency during each round is shown in Table 1. After five-time panning procedure, the fraction of unbound *E. coli *cells was significantly decreased, from about 11% to 3.9 × 10^-5^%.

**Table 1:** The binding efficiency of *E. coli *cells after each round of panning procedure on glutathione Sepharose 4B beads.

*E. coli *cells	Panning round	Input *E. coli *cells	Unbound *E. coli *cells	Elution efficiency (%)
*E. coli *cell expressing GST fragments	1	3.8×10^10^	4.2×10^9^	11.05
	2	4.2×10^10^	5.8×10^7^	0.14
	3	4.8×10^10^	5.1×10^6^	1.1×10^-2^
	4	5.6×10^10^	6.5×10^5^	1.2×10^-3^
	5	6.9×10^10^	2.7×10^4^	3.9×10^-5^

The 150 positive clones were picked up from the plates and used for screening the GST inhibitors. Following five consecutive screening procedures (consisting of screening the binding of peptides to glutathione Sepharose 4B beads, and screening the positive clones as GST inhibitors), the inhibitor efficiencies of all positive clones were compared. We found that positive clones expressing G**YW**KIKG**L**V (F1 peptide) and KW**R**NK**K**FELGLEFP**N**L (F2 peptide) can significantly inhibit GST activity. The binding efficiency of *E. coli *cells expressing F1 or F2 peptides on the glutathione Sepharose 4B beads was confirmed by an independent experiment. The fraction of *E. coli *cells expressing F1 or F2 peptides unbound to the glutathione Sepharose 4B beads was 2.3 × 10^-5^% or 1.1 × 10^-5^%, respectively, while 26.4% control *E. coli *cells remained unbound to such beads (Table 2).

**Table 2:** The binding efficiency of *E. coli *cells expressing F1 and F2 peptides on glutathione Sepharose 4B beads.

*E. coli *cells	Input *E. coli *cells	Unbound *E. coli *cells	Elution efficiency (%)
*E. coli *cells expressing F1 peptide	3.0×10^10^	6.8×10^4^	2.3×10^-5^
*E. coli *cells expressing F2 peptide	2.8×10^10^	3.2×10^4^	1.1×10^-5^
*E. coli *cells (control)	3.6×10^10^	9.5×10^9^	26.4

It has been concluded from the crystallographic study that Arg_41_, Lys_44 _and Asn_53 _of GST can interact with glutathione [16]. Here, our results show that the identified F2 peptide KW**R**NK**K**FELGLEFP**N**L contains Arg_41_, Lys_44 _and Asn_53 _(indicated in bold letters). The control peptide F4, lacking Arg_41_, Lys_44 _and Asn_53 _residues, could not bind efficiently to the glutathione Sepharose 4B beads (Table 3). Moreover, Tyr_6_, Trp_7 _and Leu_12 _of GST were shown to interact with glutathione [16], and our results show that F1 peptide G**YW**KIKG**L**V contains Tyr_6_, Trp_7 _and Leu_12 _(indicated in bold letters). Moreover, the control peptide F3, lacking Tyr_6_, Trp_7 _and Leu_12,_could not bind efficiently to glutathione Sepharose 4B beads (Table 3).

**Table 3:** The binding of synthesized peptides F1, F2, F3 and F4 to glutathione Sepharose 4B beads.

Peptide	Binding efficiency	
	
	Before elution	After elution
F1	99.6%	2.1%
F2	99.3%	1.8%
F3	1.2%	1.2%
F4	2.6%	2.5%

The binding efficiencies of different peptides were determined according to the ratio of peptide concentrations after the binding and before the binding experiments. Moreover, the peptide binding characteristics were determined again, after the peptides were eluted with the elution buffer.

Results of the screening with the use of the CAGF cloning method are consistent with the crystallographic data. The structure-function analysis has shown that GST contains one important binding site (G-site) for glutathione [1]. Experiments based on kinetic and chemical modification techniques indicated that the active site might contain either His, Cys, Trp, Arg, or Asp [17-21]. The crystal structure indicates that GST binds two molecules of glutathione sulfonate at the G-site. Several groups have investigated changes in amino acids involved in the formation of the G site of GST. The Tyr_6_, as one of the important components of the G site, is conserved in many mammalian GSTs. Tyr_6 _plays an essential role in stabilizing the thiolate anion of glutathione through hydrogen bonding. This residue was studied using site-directed mutagenesis, and when Tyr was replaced by different amino acids, GST has lost at least 90% its specific activity [22-24]. Our results with the CAGF cloning method also suggest an important function of Tyr_6 _in glutathione binding, therefore, the screening results are consistent with the crystallographic data.

### The binding characteristics of F1 and F2 peptides

To determine the binding characteristics of selected peptides (F1 and F2), an analysis of the interaction of synthesized peptides with glutathione Sepharose 4B beads was performed using the Scatchard method. The Scatchard analysis is a method of linearizing data from the binding experiment in order to determine binding capacity. The ratio of specific binding and free concentrations was plotted against specific binding concentration. The maximum binding capacity B_max _and dissociation constant K_d _of F1 and F2 peptides were determined. Our results show that there are about 1.1 F1 peptide and 1.2 F2 peptide binding sites on glutathione Sepharose 4B beads, and the disassociation constant of the F2 peptide is lover than that of the F1 peptide (Table 4).

**Table 4:** The binding of F1 and F2 peptides to glutathione.

Peptide	Binding characteristics of peptides on glutathione Sepharose 4B beads	
	
	B_max _(site)	K_d _(pM)
F1 peptide	1.1	45.6
F2 peptide	1.2	18.3

The experiments were performed according to the binding characteristics of F1 and F2 peptides to the glutathione Sepharose 4B beads. The concentrations of bound and free peptides were determined following separation of the binding complexes by centrifugation, and the free peptide concentration in the supernatant was detected by the Lowry method.

The binding efficiencies of F1-glutathione and F2-glutathione to GST were further confirmed by analysis of the binding of GST to the F1 peptide-glutathione Sepharose 4B complex and the F2 peptide-glutathione Sepharose 4B complex. The appropriate binding capacity B_max _and dissociation constant K_d _values were determined. The results show that there are about 1.2 GST binding sites for F1 peptide-glutathione Sepharose 4B complex, and 1.5 GST binding sites for F2 peptide-glutathione Sepharose 4B complex. The disassociation constant of GST on F2 peptide-glutathione Sepharose 4B complex was lower than that of GST on F1 peptide-glutathione Sepharose 4B complex (Table 5).

**Table 5:** The binding of F1 and F2 peptides to GST.

Peptide-glutathione Sepharose 4B beads	Binding characteristics of GST on peptide-glutathione Sepharose 4B beads	
	
	B_max _(site)	K_d _(pM)
F1 peptide-glutathione Sepharose 4B beads	1.2	156.3
F2 peptide-glutathione Sepharose 4B beads	1.5	114.2

The experiments were performed according to the binding characteristics of GST with F1 peptide-glutathione Sepharose 4B bead complex and F2 peptide-glutathione Sepharose 4B bead complex. The concentrations of bound and free GST were determined after separation of complexes by centrifugation, and the free GST concentration in the supernatant was determined by the Lowry method.

### The inhibitory effects of selected peptides

The synthesized peptides F1 and F2 were used in the analysis of the efficiency of inhibition of GST activity (Table 6). The inhibition efficiencies were as follows: tannic acid > cibacron blue > F2 peptide > hematin > F1 peptide > ethacrynic acid, with the use of CDNB or DCNB as the substrates. Moreover, we could not find any significant inhibition of the GST activity using control peptides F3 or F4.

**Table 6:** Effects of different inhibitors (two selected peptides F1 and F2, tannic acid, cibacron blue, hematin, and ethacrynic) on the GST activity.

Inhibitor (1 μM)	Specific GST activity (units/mg)	
	
	CDNB (1 mM)	DCNB (1 mM)
Control	2.80 ± 0.01 (100%)	4.51 ± 0.02 (100%)
F1	1.20 ± 0.01(43.1%)	2.65 ± 0.02 (58.8)
F2	0.73 ± 0.01 (26.1%)	1.49 ± 0.02 (33.2%)
tannic acid	0.16 ± 0.01 (5.6%)	0.49 ± 0.01 (10.9%)
cibacron blue	0.51 ± 0.01 (18.3%)	0.96 ± 0.01 (21.3%)
hematin	0.99 ± 0.01 (35.2%)	1.90 ± 0.02 (42.1%)
ethacrynic acid	1.54 ± 0.01 (55.1%)	3.66 ± 0.02 (81.2%)

Experiments were performed using glutathione and CDNB or DCNB as substrates. The activity was determined as described in the Methods section. Mean values ± SD for triplicate determinations are shown. A unit of GST activity was defined as the formation of 1 μmol of the product per min under the conditions of the specific assay. Specific activity is defined as the units of enzyme activity per mg of GST in the reaction system.

These results indicate that we have found an efficient GST inhibitor, the F2 peptide, which is more efficient than hematin (35.2% activity with CDNB as a substrate, 42.1% activity with DCNB as a substrate). We also found another inhibitor of this reaction, the F1 peptide, which is more efficient than ethacrynic acid (55.1% activity with CDNB as a substrate, 81.2% activity with DCNB as a substrate).

### The inhibition characteristics of selected peptides

**Figure 3:**
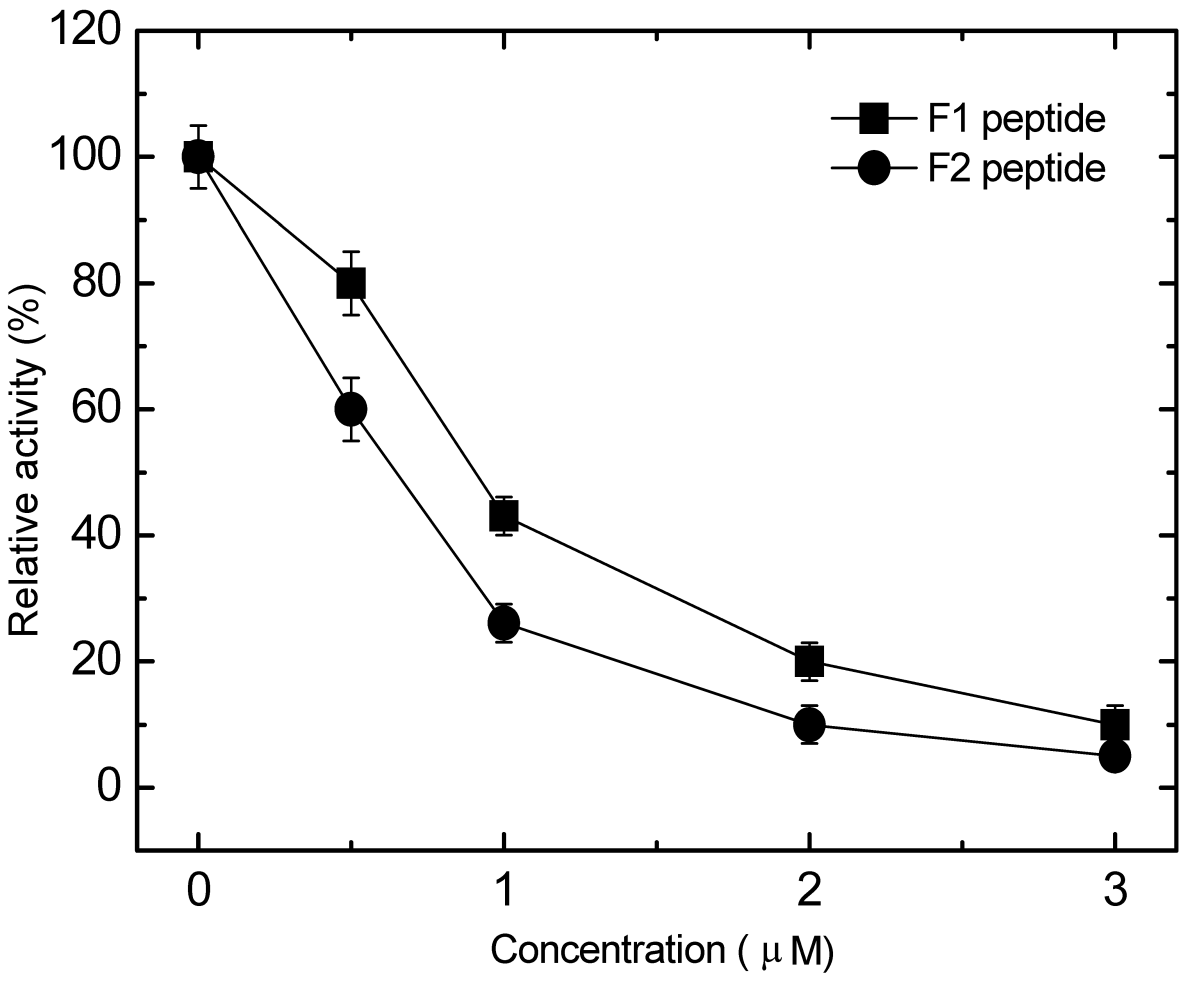
**Effects of F1 and F2 peptides on the activity of GST (μmol/mg/min)**. The activity was measured by monitoring the GST activity with different peptide concentrations, 1 mM glutathione (GSH) and 1 mM CDNB in 100 mM potassium phosphate buffer (pH6.5) at 25°C. Each point shows the mean ± SD of triplicate determinations. Relative GST activity was obtained from the ratio of GST activity in the presence of inhibitor and without inhibitor.

The inhibitory effects of selected F1 and F2 peptides on the GST-catalyzed reaction, using CDNB and glutathione as substrates, are shown in Fig. 3. Peptides F1 and F2 inhibited the reaction in a dose-dependent manner, with 50% inhibitory concentrations of 0.8 μM and 0.6 μM, respectively.

**Figure 4:**
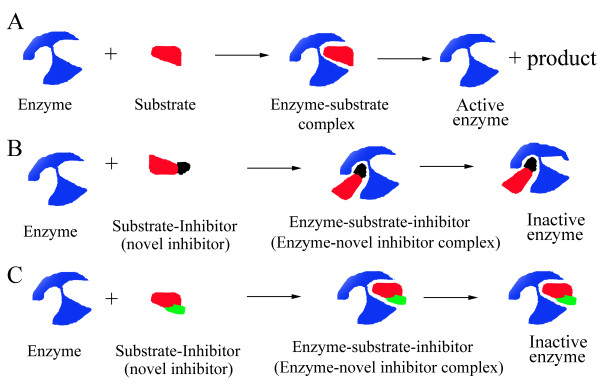
**The putative mechanisms of F1 peptide- and F2 peptide-mediated inhibition of the GST activity**. A): Binding of the enzyme (blue) and substrate (red) results in formation of the enzyme-substrate complex, and then in liberation of the final product. B): Binding of the F1 peptide (black) and substrate (red) leads to formation of a new peptide-substrate complex inhibitor, which may non-competitively inhibit the enzyme activity. C): Binding of the F2 peptide (green) and substrate (red) leads to formation of a new peptide-substrate complex inhibitor, which may competitively inhibit the enzyme activity.

To obtain information on the nature of the inhibition by F1 and F2 peptides, GST activity was measured with variable concentrations of glutathione. Here, we applied a new model in describing enzyme inhibitor (Fig. 4). It is possible that when the F1 or F2 peptide bound to the substrate glutathione, a peptide-glutathione complex was formed. Because the concentration of the peptide inhibitor was significantly lower than the substrate (glutathione) concentration, we assumed that the binding of the peptide inhibitor with glutathione will not significantly affect the substrate concentration. Hence, the newly formed peptide-glutathione complex was considered as a new inhibitor. The concentration of the peptide-glutathione inhibitor is almost equal to the peptide concentration, thus, the Michaelis-Menten equation was used in the analysis.

**Figure 5:**
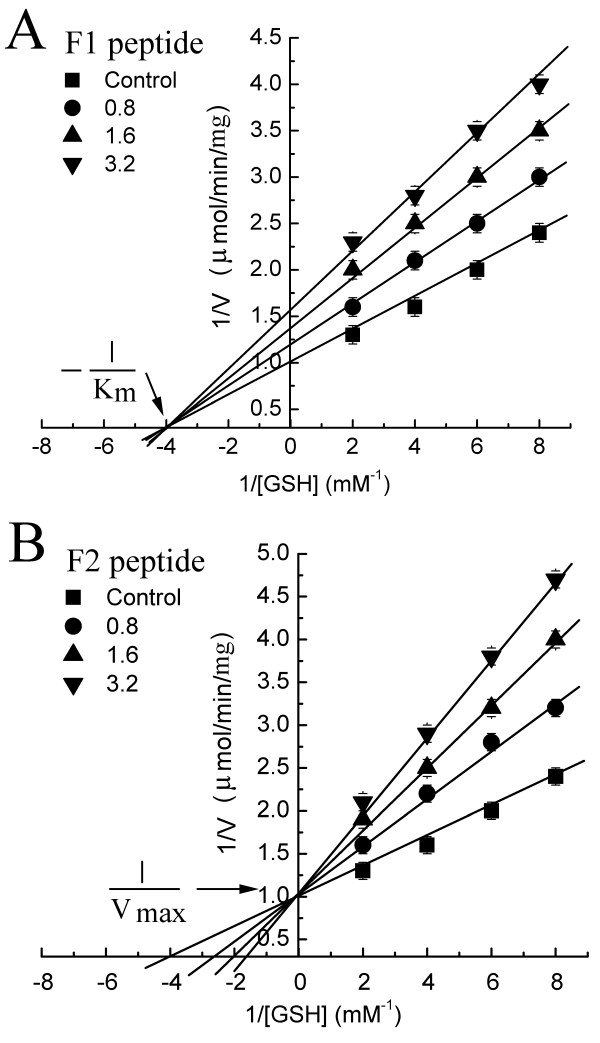
**Lineweaver-Burk plot of the GST activity with varying glutathione (GSH) concentrations**. A): The activity was measured with 1 mM CDNB, and different concentrations of GSH, and the F1 peptide. B): The activity was measured with 1 mM CDNB, and different concentrations of GSH, and the F2 peptide.

The Lineweaver-Burk plot for glutathione as the variable peptide concentration was applied to determine the types of inhibition caused by F1 and F2 peptides. The plot provides a useful graphical method for analysis of the Michaelis-Menten equation. The effects of the peptide on GST-catalyzed reaction kinetics were determined by analysis of apparent V_max_, inhibitor constant K_i _and [I]/K_i _values. Our results indicated that the F1 peptide exerted a noncompetitive inhibition in the GST-catalyzed reaction with the changing glutathione and F1 peptide concentrations (V_max _decreased while K_m _remained unchanged) (Fig. 5A). However, the F2 peptide exerted a competitive inhibition in this reaction, with the changing glutathione and F2 peptide concentrations (K_m _decreased while V_max _remained unchanged) (Fig. 5B).

The V_max_, K_i _and [I]/K_i _values for the F1 peptide were determined (Table 7). The V_max _value of the GST-catalyzed reaction was determined as 1 μmol/mg/min. However, in the presence of 0.8 μM F1 peptide inhibitor, the V_max_, K_i _and [I]/K_i _values were determined as 0.833 μmol/mg/min, 4.0 μM and 0.2, respectively. With the increasing F1 peptide concentrations, from 0.8 to 3.2 μM, the V_max _value decreased from 0.833 μmol/mg/min to 0.645 μmol/mg/min, K_i _value increased from 4.0 μM to 5.82 μM, and [I]/K_i _value increased from 0.2 to 0.55.

**Table 7:** The characterization of the F1 peptide as an inhibitor of the GST-catalyzed reaction (The V_max_, K_i _and [I]/K_i _values of the GST-catalyzed reaction in the presence of the F1 inhibit peptide were determined).

F1 peptide concentration [I] (μM)	V_max _value (μmol/mg/min)	K_i _value (μM)	[I]/K_i_
0	1.0	-	-
0.8	0.833	4.0	0.2
1.6	0.741	4.57	0.35
3.2	0.645	5.82	0.55

The K_m_, K_i _and [I]/K_i _values for the F2 peptide were also determined (Table 8). The K_m _value of the GST-catalyzed reaction was determined as 0.25 mM. However, in the presence of 0.8 μM F2 peptide inhibitor, the K_m_, K_i _and [I]/K_i _values were determined as 0.38 mM, 1.53 μM and 0.52, respectively. With the increasing F2 peptide concentrations from 0.8 to 3.2 μM, the K_m _value increased from 0.38 mM to 0.58 mM, K_i _value increased from 1.53 μM to 2.42 μM, and [I]/K_i _value increased from 0.52 to 1.32.

**Table 8:** The characterization of the F2 peptide as an inhibitor of the GST-catalyzed reaction (The V_max_, K_i _and [I]/K_i _values of the GST-catalyzed reaction in the presence of the F2 inhibit peptide were determined).

F2 peptide concentration [I] (μM)	K_m _value (mM)	K_i _value (μM)	[I]/K_i_
0	0.25	-	-
0.8	0.38	1.53	0.52
1.6	0.50	1.60	1.0
3.2	0.58	2.42	1.32

Moreover, with the changing concentrations of CDNB or DCNB, from 0.5 mM to 2 mM, the kinetics of the GST-catalyzed reaction remained similar in the reaction system containing GST, glutathione and the inhibitor (F1 peptide or F2 peptide). Therefore, we conclude that CDNB and DCNB cannot significantly affect the inhibition efficiency of F1 peptide or F2 peptide.

All these results show that effective non-competitive inhibitor F1 peptide and competitive inhibitor F2 peptide were found by using the CAGF cloning method. Although F1 and F2 peptides comprise only a small part of GST, they show significant inhibition efficiencies in the GST-catalyzed reaction.

## Discussion

The development of resistance to anticancer agents is a primary concern in cancer chemotherapy. In this light, it is obvious that the emergence of drugs, such as the GST inhibitors, able to overcome this resistance is a advancement [10,11]. Therefore, it is of special interest to develop GST inhibitors able to enhance the therapeutic index of anticancer drugs. Ethacrynic acid and quinine, which are both GST inhibitors, have been reported to reverse the resistance to melphalan and doxorubicin of cancer cell lines with increased GST expression [25]. In fact, ethacrynic acid has been used as an inhibitor of GST *in vivo*. However, first-generation GST inhibitors (e.g. ethacrynic acid) were unsuccessful in clinical trials. This might be due to its lack of specific function for GST isozyme, and propensity to react with other chemicals. In addition, there caused a number of unwanted clinical side effects. Therefore, more specific GST inhibitors may eliminate some of these undesirable features.

Here, we used the CAGF cloning method to find the GST fragments interacting with glutathione, which might be useful for the finding of GST inhibitors. We found two inhibitory peptide fragments, F1 peptide and F2 peptide. Our results revealed that F2 peptide is a potent inhibitor of the reaction with IC_50 _of 0.6 μM (Fig. 3).

**Figure 6:**
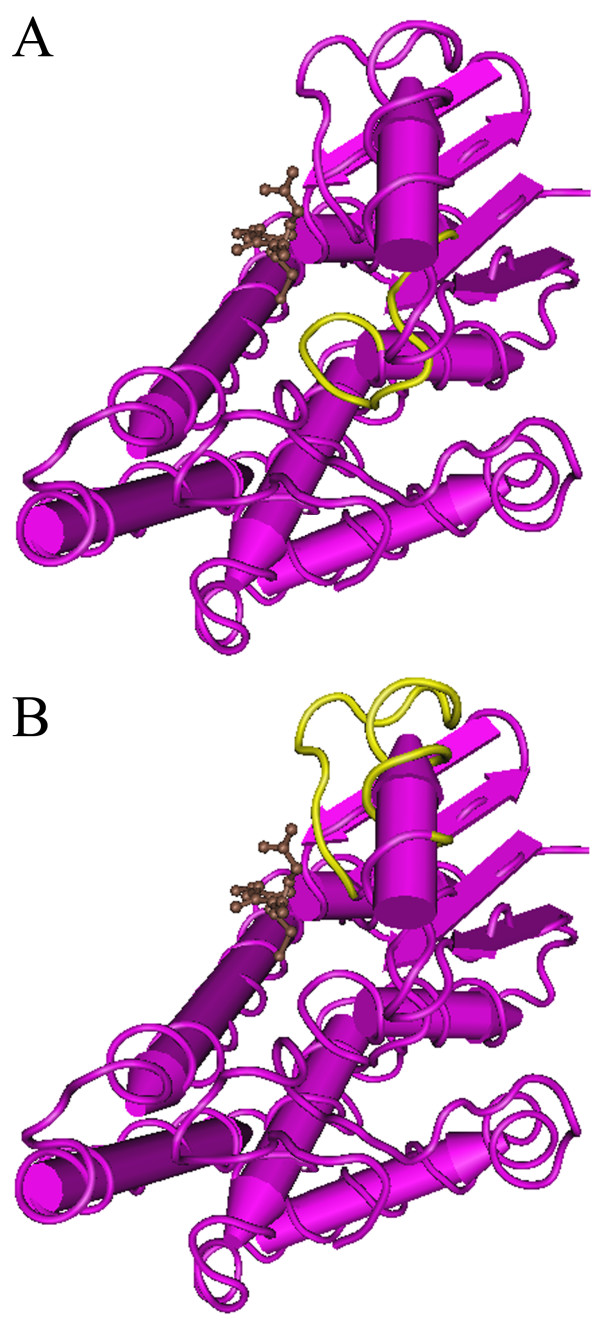
**The crystal structure of GST with putative binding sites of F1 peptide (A) and F2 peptide (B) inhibitors**. Symbols: GST (purple), glutathione (brown), selected peptide (yellow). The crystal structure of GST and glutathione complex was viewed using the protein structure data (PDB: 1m99).

The putative inhibition mechanisms of actions of F1 and F2 peptide inhibitors are shown in Fig. 4. The F1-glutathione complex was found to be a non-competitive inhibitor, suggesting that the inhibitory binding site of F1-glutathione is different from the catalytic site of GST. An analysis of the crystal structure of GST and glutathione shows that the F1 peptide is located in the interior position of the G site (Fig. 6A). It may be difficult for F1-glutathione complex to dock into the catalytic site of GST. We assume that F1-glutathione complex may be docked into non-catalytic site of GST, affect the structure of catalytic site of GST, and cause the non-competitive inhibition.

On the other hand, the F2 peptide is located in the marginal position of the G site of GST (Fig. 6B). It may be easy for F2-glutathione complex to duck into the catalytic site of GST. We assume that the F2-glutathione complex may be docked into the G site of GST. Thus, F2-glutathione may directly interfere with the catalytic site of GST and glutathione. Since the F2-glutathione causes a competitive inhibition, the F2 peptide may be a good candidate for further studies on cancer chemomodulation.

The following mechanism was used to explain the inhibitory activity of GST fragment-substrate complexes on GST-catalyzed reaction. When the F1 or F2 peptide bound to the substrate glutathione, a peptide-glutathione complex was formed. Although GST can convert glutathione into the reaction products, this enzyme cannot convert the peptide-glutathione inhibitor into the product. Thus, the binding of the peptide-glutathione to GST can inactive the enzyme activity. We speculate that peptide-glutathione occupied the functional domain or affected the functional domain of GST. Thus, GST-peptide-glutathione or GST-glutathione-peptide complex cannot catalyze the conversion of glutathione (Fig 4B and 4C). Here, the function of peptide-glutathione inhibitor is just like the substrate homologue or substrate-modifying inhibitor [26].

In summary, we have determined two glutathione-binding fragments of the GST sequence, and found that the F2 peptide, selected by the CAGF cloning method, can be considered as the inhibitor of GST. The F2 peptide is a potent inhibitor, stronger than hematin and ethacrynic acid, but weaker than tannic acid and cibacron blue. We suggest that the F2 peptide can be considered in applications against GST-induced multidrug resistance.

**Figure 7:**
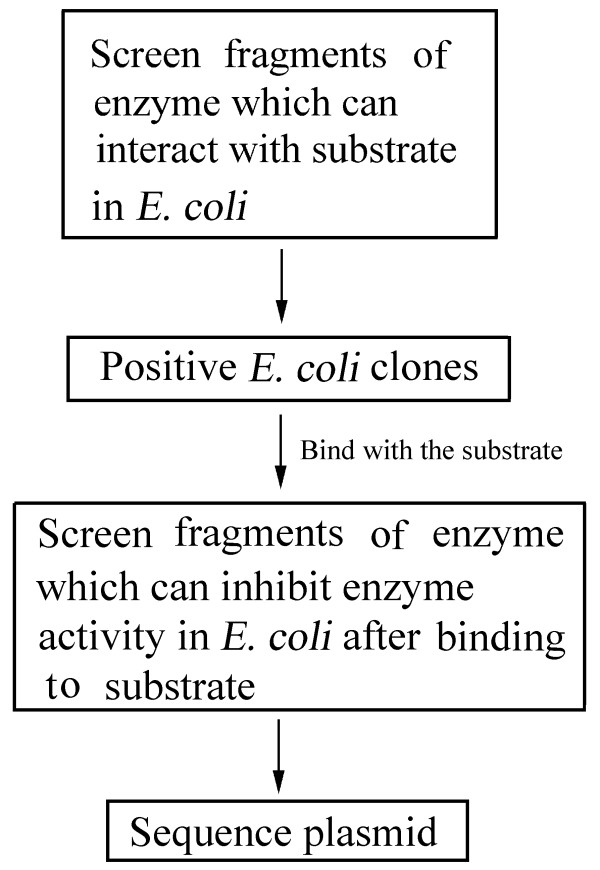
**The scheme of the novel method for finding enzyme inhibitors**.

Moreover, Fig. 7 shows the scheme of the novel method in finding enzyme inhibitors. In the first step, the enzyme fragments which can bind with the substrate were screened to find the binding peptides. In the second step, the complexes of enzyme fragments and substrate were screened to find the enzyme inhibitor. We believe that this method can be used as a common tool for finding enzyme fragments that interact with a substrate, and subsequently for finding enzyme inhibitors.

## Conclusion

In conclusion, we have successfully found a F2 peptide as GST inhibitor with the novel screening method from GST sequence. Our screening method should be useful for screening many different enzyme inhibitors.

## Methods

### Generation of the GST library

The forward primer FP1: 5' ATG TCC CCT ATA CTA GGT 3' and reverse primer RP1: 5' TCA CGA TGC GGC CGC TCG 3' were used to amplify the *Schistosoma japonicum *full-length GST gene [27] from the pGEX4T-2 plasmid DNA vector (Amersham). The amplified DNA fragments were purified with the QIAquick PCR purification kit (QIAGEN). The following reaction system was used: 1 ng pGEX4T-2 plasmid, 50 mM Tris-HCl (pH 7.8), 5 mM MgCl_2_, 10 mM 2-mercaptoethanol, 10 μg/ml BSA, 1 ng forward primer FP1 and 1 ng reverse primer RP1, 20 μM dNTP, 1 μM dideoxynucleotides (ddNTP), 2 units DNA Polymerase I (Invitrogen), and ddH_2_O to the reaction volume of 100 μl; incubation at 15°C for 30 to 60 min.

The amplified DNA library was purified by using the phenol-chloroform method, and dissolved in water. The DNA library was digested with the Exonuclease VII (Epicentre), which has a high enzymatic specificity for single-stranded DNA and exhibits both 5' → 3' and 3' → 5' exonuclease activities. This enzyme is especially useful for rapid removal of single-stranded oligonucleotide primers.

### Cloning the GST library into pFliTrx vector

The cloning of DNA library into the pFliTrx vector (Invitrogen) was performed as shown in Fig. 1. The pFliTrx was amplified using *Pfx *DNA polymerase (Invitrogen) with the forward primer FP2: 5' GGT CCG TCG AAA ATG ATC GCC CCG ATT CTG GAT 3' and the reverse primer RP2: 5' CGG ACC GCA CCA CTC TGC CCA GAA ATC GAC GAA 3'. The two-step PCR reaction was performed under the following conditions: 92°C for 2 min; then 35 cycles at 68°C for 5 min, and 92°C for 30 s. The amplified PCR product was purified by using the QIAquick PCR purification kit (QIAGEN).

The purified PCR product of linearized pFliTrx (without the fusion junction) was used to link it to the DNA library with T4 ligase. The ligation products were introduced into the *E. coli *GI826 (F^-^, *lacIq, ampC*::*Ptrp*::*c*I, Δ*fliC*, Δ*motB*, *eda*::*Tn*10) (Invitrogen).

### Screening the GST fragments which can bind to glutathione

The GST library was introduced to *E. coli *GI826 competent cells, which were then cultured in 50 ml of IMC medium (1 × M9 salts, 0.2% casamino acid, 0.5% glucose, 1 mM MgCl_2_) containing 100 μg/ml ampicillin with shaking (225–250 rpm) to saturation (OD_600 _= 3) for 15 hours at 25°C. *E. coli *cells were added to 50 ml IMC medium containing 100 μg/ml ampicillin and 100 μg/ml tryptophan for induction. The cells were grown at 25°C with shaking for 6 hours. Then, 1 ml of glutathione Sepharose 4B (Amersham) slurry and 1 ml of tryptophan-induced culture broth were added to 40 ml of the PBS buffer in a 50 ml tube, and kept at the room temperature for 30 min, centrifuged at 1,000 × *g *for 10 min at the room temperature, then resuspended in the PBS buffer, and centrifuged at 1,000 × *g *for three more times. Finally, the pellet was resuspended in 2 ml of PBS, and 500 μl of elution buffer (50 mM Tris-HCl, 10 mM reduced glutathione, pH 8.0) were added to elute the bound *E. coli *cells. The eluted *E. coli *cells were used for the next panning procedure. Following the panning procedure, 100 μl of the eluted solution was added on the RMG plates (1 × M9 salt, 0.2% casamino acid, 0.5% glucose, 1 mM MgCl_2_, 1.5% agar). The plates contained 100 μg/ml ampicillin for selection of the positive clones. Then the single positive clones from the RMG plates were picked up, and 150 single clones were used for screening the GST inhibitors.

### Screening of GST fragments which can inhibit the GST activity

150 single positive clones (grown on the RGB plates), that could tightly bind to the glutathione Sepharose 4B, were picked up from the plates, and cultured separately in 50 ml of IMC medium containing 100 μg/ml ampicillin for 15 hours at 25°C, then induced with 100 μg/ml tryptophan for 6 hours. *E. coli *cells were washed with the PBS buffer for three times at 4°C, and suspended in the 5 ml PBS solution, respectively.

Recombinant *S. japonicum *GST, glutathione, tannic acid, cibacron blue, hematin, ethacrynic acid, 1,2-dichloro-4-nitrobenzene (DCNB) and 2,4-dinitrochlorobenzene (CDNB) were purchased from Sigma-Aldrich, and used to measure the GST activity.

To measure the GST activity, glutathione and CDNB solutions were added (to final concentration of 1 mM) to 100 μl of *E. coli *cell suspension (10^8 ^cells). Then, GST solution was added (the cell suspension without glutathione was used as the control). The GST activity was measured by using the spectrophotometric assay [28].

The single clones, which can produce the GST inhibitory peptide, were selected again, and the plasmid DNA was extracted for determination of inserted sequences. The whole screening procedures were performed five times. Finally, plasmid DNAs were extracted from *E. coli *cells expressing inhibitory peptides, and sequenced.

### Analysis of the binding of peptides to glutathione

The binding characteristics of selected peptides to glutathione were determined according to the analysis of binding of synthesized peptides to the glutathione Sepharose 4B beads. The amount of glutathione in the glutathione Sepharose 4B beads was estimated according to the assumption (according to the manufacturere's information) that there are about 200–400 μmol glutathione/g dried beads. An average value of 300 μmol glutathione/g dried beads was used to calculate the amount of glutathione in the glutathione Sepharose 4B beads. In our experiments, appropriate amount of wet glutathione Sepharose 4B beads (equal to 1 mg dry beads) was added to a 1.5 ml Eppendorf tube, and different amounts of synthesized peptides were added into the tube. After binding for 10 min at 37°C, the binding complexes were separated by centrifugation (12,000 × *g *for 10 min) and concentrations of bound and free peptides were determined by using the Lowry method [29]. Scatchard analysis was used to determine the K_d _and B_max _values. B_max _means the maximum binding sites of synthesized peptide on glutathione Speharose 4B beads (μmol peptide/μmol glutathione). K_d _is a dissociation constant (pM). Thus, a low K_d _value indicates a high affinity.

### Analysis of the binding of peptide-glutathione complex to GST

The binding of selected peptide-glutathione complexes with GST were determined on the basis of analysis of binding of GST to the peptide-glutathione Sepharose 4B bead complex. Wet glutathione Sepharose 4B beads (equal to 1 mg dry wet) was added into a 1.5 ml Eppendorf tube for binding to peptides. After the binding of peptides to glutathione Sepharose 4B beads at 37°C for 10 min, the unbound peptides were washed out. Then, different amounts of GST were added into the tube. After binding for 10 min at 37°C and separation of the bound complexes by centrifugation, the amounts of bound and free GST were determined by the Lowry method [29]. Scatchard analysis was used to determine the K_d _and B_max _values. B_max _means the maximum binding site of GST with the peptide on peptide-glutathione Speharose 4B beads (μmol GST/μmol peptide-glutathione). K_d _is a disassociation constant (pM).

### Enzyme inhibition assay

When the screening experiments were performed, four peptides were synthesized to analyze their inhibition efficiencies. F1 peptide (G**YW**KIKG**L**V, yield: 25.3 mg), F2 peptide (KW**R**NK**K**FELGLEFP**N**L, yield: 28.1 mg), F3 peptide (GKIKGV, yield: 2.2 mg) and F4 peptide (KWNKFELGLEFPL, yield: 1.9 mg) were obtained from the Invitrogen (Custom Peptide Synthesis, with the purity > 95%). Recombinant *S. japonicum *GST (~40 units/mg), glutathione, tannic acid, cibacron blue, hematin, ethacrynic acid, 1,2-dichloro-4-nitrobenzene (DCNB) and 2,4-dinitrochlorobenzene (CDNB) were from Sigma-Aldrich Co. The inhibition studies were carried out according to the previously described method [28] at 25°C using glutathione (1 mM) and CDNB (1 mM) or DCNB (1 mM) as substrates. The inhibitors (tannic acid, cibacron blue, hematin, ethacrynic acid or the synthesized peptides) were added to the reaction mixture and GST activity was determined.

The peptide concentration resulting in 50% inhibition (IC_50_) was determined from a plot of remaining activity versus peptide concentration. Protein concentration was measured according to the Lowry method [29]. Enzyme inhibitory kinetic studies were carried out using various concentrations of glutathione and CDNB and different concentrations of synthetic peptides (0.8, 1.6, 3.2 μM).

## Abbreviations

CAGF: covering all gene fragments; GST: glutathione transferase; CDNB: 2,4-dinitrochlorobenzene; DCNB: 1,2-dichloro-4-nitrobenzene; CLL: chronic lymphocytic leukemia.

## Competing interests

The authors declare that they have no competing interests.

## Authors' contributions

All authors have contributed to the content of the article, and all authors approved the final version.
